# Recent increase of genetic diversity in *Plasmodium vivax *population in the Republic of Korea

**DOI:** 10.1186/1475-2875-10-257

**Published:** 2011-09-07

**Authors:** Hajime Honma, Jung-Yeon Kim, Nirianne MQ Palacpac, Toshihiro Mita, Wonja Lee, Toshihiro Horii, Kazuyuki Tanabe

**Affiliations:** 1 Department of Molecular Protozoology, Research Institute for Microbial Diseases, Osaka University, Suita, Osaka, Japan; 2 Laboratory of Malariology, Research Institute for Microbial Diseases, Osaka University, Suita, Osaka, Japan; 3 Division of Malaria and Parasitic Diseases, National Institute of Health, Korea Center for Diseases Control and Prevention, Osong, Korea; 4 Department of International Affairs and Tropical Medicine, Tokyo Women's Medical University School of Medicine, Shinjuku-ku, Tokyo, Japan

## Abstract

**Background:**

The reemergence of *Plasmodium vivax *in South Korea since 1993 represents a serious public health concern. Despite the importance in understanding genetic diversity for control strategies, however, studies remain inconclusive with the general premise that due to low rate of malaria transmission, there is generally low genetic diversity with very few strains involved. In this study, the genetic diversity and population structure of *P. vivax *in South Korea were explored by analysing microsatellite polymorphism.

**Methods:**

Sequences for 13 microsatellite loci distributed across the twelve chromosomes of *P. vivax *were obtained from 58 South Korean isolates collected during two sampling periods, namely 1997-2000 and 2007. The sequences were used for the analysis of expected heterozygosity and multilocus genotype diversity. Population structure was evaluated using STRUCTURE version 2.3.2. Linkage disequilibrium was also analysed to investigate the extent of outbreeding in the *P. vivax *population.

**Results:**

Mean expected heterozygosity significantly increased from 0.382 in 1997-2000 to 0.545 in 2007 (*P *< 0.05). The number of multilocus genotypes was 7 and 27; and genotype diversity was statistically significant (*P *< 0.01) at 0.661 and 0.995 in 1997-2000 and 2007, respectively. Analysis by STRUCTURE showed a more complex population structure in 2007 than in 1997-2000. Linkage disequilibrium between 13 microsatellites, although significant in both time points, was notably lower in 2007.

**Conclusions:**

The present microsatellite analysis clearly showed recent increase of genetic diversity and recent relaxation of the strong population structure observed in 1997-2000. These results suggest that multiple genotypes not present previously recently migrated into South Korea, accompanied by substantial outbreeding between different genotypes.

## Background

Of the five human malaria parasites, *Plasmodium vivax *is the most prevalent in Asia, Melanesia, the Middle East, South and Central America, accounting for 70-80 million cases annually [1], with 2.6 billion people at risk of infection [2]. Despite modest gains in *Plasmodium falciparum *control, the global burden of *P. vivax *remains underestimated [3] and emergence of drug resistant *P. vivax *makes the control of vivax malaria more difficult than before [4,5]. In the Republic of Korea (South Korea), vivax malaria was eradicated once in the late 1970s [6]. However, indigenous malaria has reemerged since the first infection of a soldier who had never been abroad was confirmed in 1993 in the Demilitarized Zone (DMZ) between South and North Korea [7]. Thereafter, vivax malaria infection has spread to civilians and the number of cases from 2000 has fluctuated between 864 and 4,142 [8]. Thus, vivax malaria has become endemic again and constitutes a serious public concern in South Korea. Meanwhile, in North Korea, vivax malaria is more prevalent than in South Korea and the number of cases has fluctuated between 7,436 and 296,540 from 2000 [6,9].

Genetic diversity and population structure of *P. vivax *have a significant impact on malaria transmission, spread of drug-resistance and the acquisition of protective immunity against malaria. Studies of the parasite population diversity have practical significance for the strategic development and deployment of control measures [10]. Limited studies on antigen-encoding genes such as merozoite surface protein-1 (MSP-1), circumsporozoite surface protein (CSP) and merozoite surface protein-3α (MSP-3α) in South Korea have previously shown that the diversity of those genes was low in 1990s, but increased after 2000 [9,11]. An increase in antigen diversity potentially affects the acquisition of protective immunity against vivax malaria. It is therefore important to determine whether the observed recent increase of the diversity of antigen-encoding genes is caused by human immune pressure or not. Since these antigens are exposed to the human immune system, diversity in the antigen-encoding loci can be attributed to not only to natural selection by immune pressure but also to the parasite's population history [12]. Hence, it remains uncertain whether the low diversity observed in the antigen-encoding loci resulted from selective pressure imposed by the host immune system or due to a demographic change in the local parasite population. In contrast to polymorphic antigen genes, microsatellite markers are selectively neutral and highly polymorphic, and, thus, are suitable for the assessment of genetic diversity resulting from demography at the genome-wide level. Recently, microsatellite markers have been widely used to analyse genetic diversity of *P. vivax *populations [13-18]. In the present study, multilocus microsatellite analysis was conducted to elucidate genetic diversity and population structure of *P. vivax *in South Korea. Results showed drastic changes in genetic diversity and population structure between 2000 and 2007.

## Methods

### Parasite samples

Blood samples were collected from indigenous *P. vivax*-infected patients between 1996 and 2007 near the DMZ in the Republic of Korea [9]. In this study, two groups classified according to the sampling year were analysed: one group of 29 samples collected between 1997 and 2000, and the other from 29 samples collected in 2007. Informed consent was obtained from all patients before blood sampling. The study was approved by Korea's National Institute of Health, and Center for Disease Control and Prevention.

### Preparation of parasite DNA

*Plasmodium vivax *DNA were extracted from 200 μL blood using proteinase K digestion technique and followed either by purification with phenol-chloroform or QIAamp^® ^DNA Mini Kit (Qiagen) following the manufacturer's instructions.

### PCR and sequencing analysis for genotyping

Thirteen validated polymorphic microsatellite markers, 1.501, 3.27, 3.502, MS3, MS15, MS5, MS9, MS16, MS20, MS6, MS8, MS10 and 14.297 [15,19], distributed across the twelve chromosomes of *P. vivax *(Additional file 1) were used for analysis. Microsatellites were sequenced to achieve higher-resolution analysis rather than the widely employed fragment-length measurements. Besides, *P. vivax *microsatellites used in this study contain relatively long and somewhat degenerating repeating units, which cannot be satisfactorily assessed by fragment-length measurements. For example, in MS20 two alleles observed with the same length, (GAA)_5_(CAA)_3 _and (GAA)_6_(CAA)_2_, would be indistinguishable from each other by fragment-length measurement. Primers for each locus were newly designed in order to perform polymerase chain reaction (PCR) and subsequent sequencing (Additional file 1). PCR reactions were performed in 20 μL reaction mixture containing 1× LA PCR™ Buffer, 2.5 mM MgCl_2_, 0.4 mM dNTP, 0.2 μM of each primer solution, 0.05 U LA Taq^® ^(Takara Bio Inc.) and 1 μL DNA template. The reaction protocol was as follows: 1 cycle at 93°C for 1 min; 40 cycles at 93°C for 20 s, 62°C for 4 min; and 1 cycle at 72°C for 10 min. The nucleotide sequence of each locus was determined directly from the PCR products using BigDye Terminator v3.1 Cycle Sequencing Kit (Applied Biosystems) on an Applied Biosystems 3130 Genetic Analyzer (Applied Biosystems) following the manufacturer's instructions. An allele for each locus corresponds to the number of repeat-motifs. Genotype is defined as the unique combination of alleles across the 13 microsatellite loci examined for this study.

### Data analysis

Expected heterozygosity (*h*_E_) of each locus was calculated by using the formula *h*_E _= {*n*/(*n*-1)}(1-∑*pi*^2^), where *n *is the number of isolates analysed and *pi *is the frequency of the i^th ^allele in the population. Variance (*V*) of *h*_E _was calculated by using the formula *V *= 2/{*n*(*n*-1)}[a+b], where a = 2(*n*-2){∑*pi*^3^-(∑*pi*^2^)^2^} and b = ∑*pi*^2^-(∑*pi*^2^)^2 ^[20]. The standard error (SE) of *h*_E _was defined as the square root of *V*. Mean expected heterozygosity (*H*_E_) of all loci and the standard error were also calculated. The statistical significance of the mean differences between expected heterozygosity values was determined by *t*-test as described by Nei, 1987 [21]. Genotype diversity (*gd*) was calculated similar to expected heterozygosity.

The standardized index of association (*I*^*S*^_*A*_) was calculated to test the presence of overall multilocus linkage disequilibrium [22]. This test compares the variance (*V_D_*) of the number of alleles shared between all pairs of haplotypes observed in the population (*D*) with the variance expected under random association of alleles (*V_E_*) as follows: *I^S^_A _*= (*V_D_*/*V_E_*-1)/(*r*-1), where *r *is the number of loci analysed. *V_E _*is derived from 100,000 simulated data sets in which alleles were randomly reshuffled among haplotypes. Linkage disequilibrium (LD) is significant if *V_D _*is > 95% of the values derived from the reshuffled data sets. Data were analysed using LIAN version 3.5 software [23]. LD for all pairs of loci was also tested using the Genepop version 4.1 [24] under the following Markov chain parameters: dememorization number = 20,000, number of batches = 500 and number of iterations per batch = 10,000.

Population structure was evaluated using the STRUCTURE version 2.3.2 software [25]. This Bayesian model-based clustering method assigns samples to *K *populations according to allele frequencies of each locus. The program was run 20 times at each of 10 different *K *values (1-10) with a period of 50,000 burn-in steps followed by 100,000 iterations. The admixture model with independent allele frequencies was used in all analyses. The number of populations was inferred by plotting the log probability of the data [Ln P(D)] for each *K *value. Also, in order to determine the most appropriate number of *K*, Δ*K *was calculated as described by Evanno *et al *[26]. Illustrations were generated using the DISTRUCT program [27].

## Results

### Genetic diversity

Microsatellite sequences from 29 isolates in 1997-2000 (Group 1) and another 29 in 2007 (Group 2) were obtained in this study (*n *= 58 isolates in total). There were two cases in the 1997-2000 group where three sequences could not be obtained. All microsatellite loci were polymorphic, exhibiting three to six alleles per locus (Table 1 and Additional file 2). The total number of alleles detected was 60 in the two groups and the number increased from 33 in 1997-2000 to 50 in 2007. Of the 60 alleles, 23 alleles were found in both sampling periods, while 10 and 27 alleles were unique in 1997-2000 and in 2007, respectively, suggesting drastic change in microsatellite alleles between 1997-2000 and 2007. In eight loci, the number of alleles increased in 2007, and in other loci frequency remained similar with one exception, locus 1.501, where allele number was low in 2007 as compared to 1997-2000. Expected heterozygosity (*h*_E_) for each locus ranged from 0.069 to 0.548 for 1997-2000, and from 0.133 to 0.778 for 2007 (Table 1). All the loci, except 1.501 and MS9, showed higher *h*_E _in 2007 than in 1997-2000. These exceptions underscore the importance of using several markers to characterize genetic diversity of a population. Mean expected heterozygosity (*H*_E_) was 0.382 ± 0.061 and 0.545 ± 0.063 in 1997-2000 and 2007, respectively (Table 1). The difference between the two sampling periods was statistically significant (*P *= 0.023), indicating a recent increase in the genetic diversity of the *P. vivax *population in South Korea.

**Table 1:** Expected heterozygosity at the different microsatellite loci of *P. vivax *populations in the Republic of Korea

		1997-2000		2007	
			
Chromosome	Locus	No. of alleles	***h***_**E **_**± SE**	No. of alleles	*h*_E _± SE
1	1.501	3	0.542 ± 0.046	2	0.192 ± 0.090
3	3.27	3	0.530 ± 0.005	4	0.665 ± 0.051
3	3.502	2	0.488 ± 0.049	4	0.751 ± 0.035
4	MS3	2	0.069 ± 0.063	2	0.133 ± 0.081
5	MS15	3	0.530 ± 0.054	5	0.692 ± 0.053
6	MS5	2	0.476 ± 0.057	6	0.727 ± 0.070
8	MS9	3	0.512 ± 0.063	3	0.362 ± 0.105
9	MS16	3	0.548 ± 0.045	5	0.778 ± 0.040
10	MS20	2	0.069 ± 0.063	2	0.296 ± 0.093
11	MS6	3	0.521 ± 0.061	5	0.751 ± 0.051
12	MS8	2	0.069 ± 0.063	5	0.594 ± 0.077
13	MS10	2	0.069 ± 0.063	2	0.502 ± 0.040
14	14.297	3	0.542 ± 0.046	5	0.645 ± 0.065

	Overall	33	0.382 ± 0.061	50	0.545 ± 0.063

A total of 33 multilocus genotypes, which were defined as the unique association of alleles in the 13 microsatellite loci, were detected: seven in 1997-2000 and 27 in 2007 with one genotype (no. 25) found in both groups (Table 2). Two of the seven genotypes (no. 25 and 30) comprised 81.5% (22/27) in 1997-2000. Remarkably, for 2007, most genotypes were found only once, except for no. 7 and 8. These suggest a drastic change in genetic diversity between the two groups. Genotype diversity (*gd*) was 0.661 ± 0.005 for 1997-2000, and 0.995 ± 0.000 for 2007 (*P *= 0.00004). Hence, the diversity of multilocus genotypes appears to have significantly increased between 2000 and 2007.

**Table 2:** Microsatellite genotype frequency of *P. vivax *populations in South Korea

	Locus†													1997-2000 (*n *= 27*)		2007 (*n *= 29)	
	
GN‡	1	3a	3b	4	5	6	8	9	10	11	12	13	14	*n*	%	*n*	%
1	11	7	4	8	9	12	5	44	11	9	26	32	15	1	3.7	0	0
2	3	14	4	7	13	16	6	24	5	6	10	38	10	0	0	1	3.4
3	3	14	4	9	13	16	7	24	6	6	10	38	13	0	0	1	3.4
4	3	14	4	9	34	9	6	24	6	2	55	38	10	0	0	1	3.4
5	3	14	5	9	12	16	6	66	6	6	55	39	13	0	0	1	3.4
6	3	14	5	9	13	16	6	66	5	6	10	39	10	0	0	1	3.4
7	3	14	5	9	13	16	6	66	6	6	54	39	13	0	0	2	6.9
8	3	14	5	9	13	16	6	66	6	6	55	39	13	0	0	2	6.9
9	3	14	5	9	13	16	6	66	6	6	56	39	13	0	0	1	3.4
10	3	14	5	9	13	16	6	66	6	9	55	39	13	0	0	1	3.4
11	3	14	5	9	34	9	6	24	5	6	55	39	13	0	0	1	3.4
12	3	17	11	9	12	14	6	21	6	10	55	38	14	0	0	1	3.4
13	3	17	11	9	12	14	6	21	6	9	10	38	9	0	0	1	3.4
14	3	17	11	9	12	14	6	82	6	9	10	38	13	0	0	1	3.4
15	3	17	11	9	12	14	8	82	6	9	10	38	13	0	0	1	3.4
16	3	17	11	9	12	9	6	82	6	2	10	38	10	0	0	1	3.4
17	3	17	11	9	13	16	6	66	6	6	10	39	13	0	0	1	3.4
18	3	17	11	9	13	16	6	82	6	9	58	39	13	0	0	1	3.4
19	3	17	11	9	34	18	7	21	6	7	10	38	10	0	0	1	3.4
20	3	17	13	9	12	14	8	82	6	9	10	38	13	0	0	1	3.4
21	3	17	13	9	12	16	6	66	6	6	10	39	13	0	0	1	3.4
22	3	19	4	9	12	17	6	21	6	10	10	38	12	0	0	1	3.4
23	3	20	13	7	33	12	7	63	5	2	10	38	10	0	0	1	3.4
24	3	20	13	9	12	12	6	21	5	10	55	38	10	0	0	1	3.4
25	3	20	13	9	12	12	8	63	6	7	10	38	10	14	51.9	1	3.4
26	5	17	11	9	12	12	6	21	6	9	10	38	10	0	0	1	3.4
27	5	17	5	9	11	16	6	21	6	10	10	38	12	0	0	1	3.4
28	5	19	13	9	11	12	6	21	6	10	10	38	12	1	3.7	0	0
29	5	19	4	9	11	12	8	21	6	7	10	38	12	1	3.7	0	0
30	5	19	4	9	11	17	6	21	6	10	10	38	12	8	29.6	0	0
31	5	19	4	9	11	17	8	21	6	7	10	38	12	1	3.7	0	0
32	5	19	4	9	11	18	6	21	6	10	10	38	12	0	0	1	3.4
33	5	20	13	9	12	17	6	21	6	10	10	38	12	1	3.7	0	0

Total number of genotypes														7		27	
Genotype diversity (*gd*)														0.661 ± 0.074		0.995 ± 0.011	

A total of 33 unique combinations of alleles (i.e. multilocus genotypes) across the 13 microsatellite loci were detected in this study. An allele for each locus corresponds to the number of repeat-motifs. Multilocus genotype frequencies in each sample group are shown.

†Locus names are displayed: locus 1, 3a, 3b, 4, 5, 6, 8, 9, 10, 11, 12, 13 and 14 correspond to 1.501, 3.27, 3.502, MS3, MS15, MS5, MS9, MS16, MS20, MS6, MS8, MS10 and 14.297, respectively.

*Two samples from 1997-2000 isolates that had missing loci were excluded.

‡GN, genotype no.

### Linkage disequilibrium

**Figure 1:**
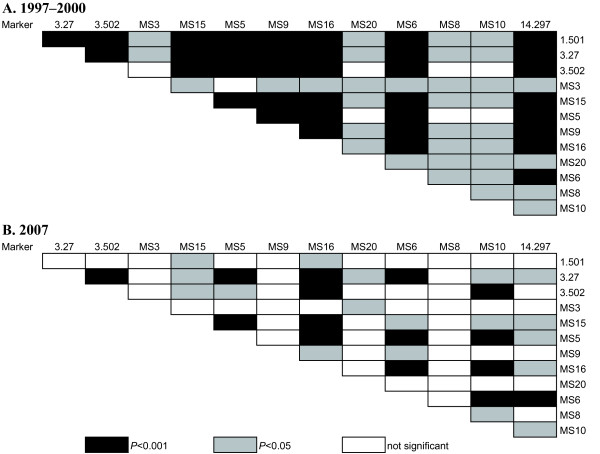
**Linkage disequilibrium between pairs of 13 microsatellite markers in *P. vivax *populations**. Linkage disequilibrium in 1997-2000 (A) and 2007 (B) from the Republic of Korea. Light and dark grey partitions denote significant linkage disequilibrium at 5% and 0.1%, respectively.

The overall multilocus linkage disequilibrium assessed by LIAN showed significant LD in 1997-2000 and 2007. LD was stronger in earlier than in recent sampling period: *I^S^_A _*= 0.5565, *P *< 0.001 for 1997-2000 and *I*^*S*^_*A *_= 0.1813, *P *< 0.001 for 2007. Linkage disequilibrium for each pair of microsatellite loci by Genepop further supported the difference between 1997-2000 and 2007. LD was significant in 70 pairs of the 78 possible pairs (90%) for 1997-2000, while it was significant in 33 of all possible pairs (42%) for 2007 (Figure 1). The microsatellite loci used in this study were located on different chromosomes, other than 3.27 and 3.502, which are both located on chromosome 3, separated by 42 kilobases. These results, thus, indicate that the genetic shuffling of different chromosomes occurred rarely during sexual reproduction in the mosquito vector in 1997-2000, while it was substantial in 2007, suggesting a high level of inbreeding in 1997-2000 and substantial outbreeding in 2007. Strong linkage disequilibrium observed between 3.27 and 3.502 indicate that meiotic recombination did not occur between these loci in the same chromosome in 1997-2000 and 2007.

### Assignment analysis to infer population structure

**Figure 2:**
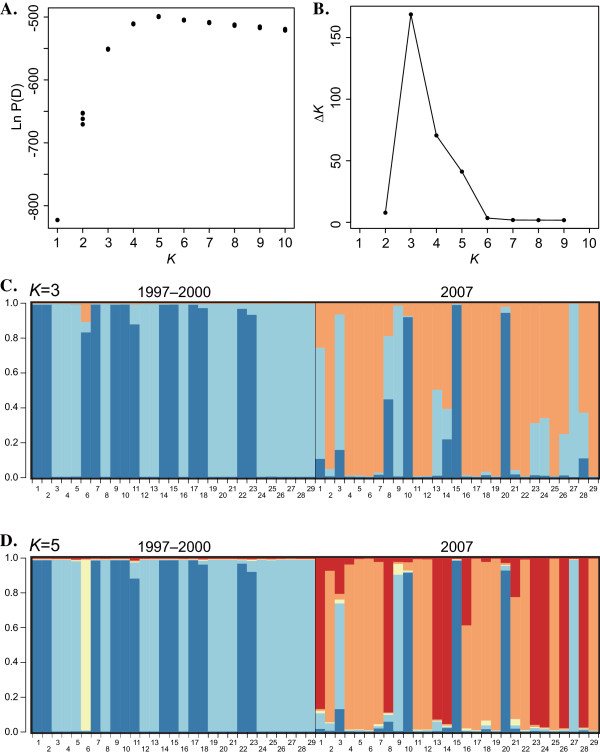
**Drastic change in the population structure of *P. vivax *between 1997-2000 and 2007**. The population structure of *P. vivax *in the Republic of Korea was inferred using STRUCTURE analysis and plots were drawn using DISTRUCT. (A) Plot of log probability of data [Ln P(D)] against the number of populations tested (*K*). (B) Plot of Δ*K *(the rate of change in the log probability of the data between successive *K *values) against the number of *K*. An individual parasite isolate is represented by a single vertical bar, which is partitioned into three (C) or five (D) segments. Sample numbers are shown along the horizontal axes. Each segment represents one population, and the colour of the segment represents the estimated membership fraction in the individual parasite assigned to *K *clusters.

Analysis by STRUCTURE is shown in Figure 2. The highest Ln P(D) which suggests the strongest statistical support was observed at *K *= 5 (Figure 2A). The most appropriate estimate of *K*, the highest Δ*K*, was observed at *K *= 3 (Figure 2B). For these reasons, these two *K *values were adopted. In Figures 2C and 2D, an individual isolate is represented by each vertical bar, in which segments (represented by colours) are proportionally partitioned according to the estimated membership fraction assigned to *K *clusters. In both *K *values, population was highly distinctive between 1997-2000 and 2007. Isolates from 1997-2000 were mainly derived from two populations, with a majority of isolates assigned to a single cluster. These indicate strong population structure in 1997-2000 and suggest that outbreeding between different populations rarely occurred. Clustering patterns at *K *= 5 (but not *K *= 3*) *suggests that isolate no. 6 in 1997-2000 was a clearly distinct from others. Isolates from 2007 were mainly derived from three clusters at *K *= 3 and four clusters at *K *= 5. Of note, one cluster at *K *= 3 (shown in orange) and two clusters at *K *= 5 (shown by red and orange colours) predominated in 2007, but were rare in 1997-2000. Clusters that predominated in 1997-2000 were minor in 2007. Some isolates in 2007 (e.g., no. 1, 3, 8 and 10) were assigned to multiple clusters (Figure 2D), which composed those clusters that predominated in 1997-2000 and those that newly appeared in 2007. These results indicate that a dramatic change occurred in the *P. vivax *population structure: the strong population structure found in 1997-2000 was relaxed and became more complex in 2007; and, likewise, substantial outbreeding among different parasite genotypes in 2007 is suggested.

## Discussion

This study is the first to report microsatellite diversity of the *P. vivax *population in South Korea. The present results obtained with 13 microsatellite markers across 12 chromosomes revealed a higher genetic diversity in 2007 than in 1997-2000. Consistent with previous limited studies showing a recent increase in antigen diversity, the study further support changes in *P. vivax *isolates from South Korea. Analysis of South Korean isolates before 2000 using antigen-encoding genes such as Duffy-binding protein [28], PvCSP [9], PvMSP-3α [11], and AMA-1 [29] infers only two alleles whereas genetic diversity of these antigens remarkably increased after 2001 [9,11]. The present study shows only two major multilocus microsatellite genotypes that comprise the two major *P. vivax *populations in 1997-2000, while numerous genotypes appeared in 2007. It is surmised that the recent increase in *P. vivax *genetic diversity was displayed at the genome wide level, and suggest that increased antigen diversity was not driven, if any, by selective pressure on antigen genes. While it is of note that the *P. vivax *genetic diversity in South Korea has increased in recent years (*H*_E _= 0.55 in 2007), diversity is still low as compared to levels obtained from other geographic areas with the exception of Peru: thus, *H*_E _= 0.71-0.86 in Brazil, Colombia, India, Laos, Thailand, Sri Lanka, Vietnam, Ethiopia, and Myanmar [13-15,17,19], *vs H*_E _= 0.44 - 0.69 in Peru (Table 3) [18]. However, although the South Korean *P. vivax *populations appear to have relatively low genetic diversity, direct comparisons of *H*_E _with other parasite populations from different geographical areas must be treated with caution especially when the number of samples used is substantially different. In this study, sample number is somewhat small (*n *= 58) as compared with other geographical areas (e.g., 159 in Peru [18]; 140, 167 and 118 in Sri Lanka, Myanmar and Ethiopia, respectively [14]). Further investigations using more samples should be performed to confirm a low genetic diversity in *P. vivax *population in South Korea.

**Table 3:** Microsatellite expected heterozygosity (*H*_E_) of *P. vivax *populations from South Korea vs other geographic areas

Geographic area	Study year	***H***_**E**_	Reference
Brazil	1999-2005	0.71-0.80	13

Colombia	2001-2003	0.79	15
India	2003-2004	0.72	
Thailand	1992-1998	0.76	
Laos	2001-2003	0.75	

Sri Lanka	2004-2005	0.79	19

Ethiopia	2006-2008	0.752	14
Myanmar	2007	0.845	
Sri Lanka	2003-2008	0.861	

Vietnam	1999-2000	0.86	17

Peru	2006-2008	0.44-0.69	18

South Korea	1997-2000	0.382	this study
	2007	0.545	

Two mechanisms can be proposed to account for the observed increase in the genetic diversity of *P. vivax *population in South Korea. One is the accumulation of microsatellite mutations in the population, and the other is migration of novel parasite populations from other geographic areas into South Korea. Since the mutation rate(s) of *P. vivax *microsatellite is unknown, accumulation of mutation cannot be completely excluded. However, the present study does reveal the appearance of very many novel alleles at almost all the microsatellite loci examined. Previous studies have also reported rapid increase in genetic diversity in antigen-encoding genes [9,11]. It is very unlikely that in a short period (between 2000 and 2007, probably six generations of the parasite life cycle in South Korea), many novel alleles accumulate simultaneously at several microsatellite and antigen loci. The idea of migration of multiple different parasite genotypes from other geographic areas into South Korea is, therefore, favoured. Haplotype network analysis based on the parasite mitochondrial genome infers a genealogical origin in southern China for *P. vivax *populations collected in 1999 and one in 2002 [30]. Further investigations on genetic diversity of *P. vivax *in neighbouring geographic areas can elucidate and verify the population mechanism.

The rate of inbreeding also contributes to the extent of genetic diversity in *P. vivax *populations. In this study, the extent of inbreeding inferred from linkage disequilibrium was reduced in 2007, compared with that in 1997-2000; although overall linkage disequilibrium was significant in both sampling groups. These results indicate very strong linkage disequilibrium, leading to a high inbreeding rate in the parasite population in 1997-2000, whereas relatively weak linkage disequilibrium, leading to elevation of outbreeding levels in 2007. Analysis by STRUCTURE (Figure 2C and 2D) supports the increased outbreeding in 2007. It is thus concluded that the recent diversification of South Korean *P. vivax *population resulted partly from an increase in the level of outbreeding among different parasite genotypes.

Moving forward, molecular tools and techniques can elucidate parasite genetic diversity and population structure that can help malaria control efforts and understand mechanisms of pathogenicity and drug resistance. It is generally believed in a parasite population with a high genetic diversity, protective immunity against malaria is slow to develop. There is accumulating evidence, which suggest that *P. vivax *is more virulent than previously thought in tropical endemic areas [5], where genetic diversity is generally high (Table 3). This raises the potential risk of severe malaria in South Korea if genetic diversity further increase to a level observed in tropical endemic areas. Recently, drug-resistant *P. vivax *has been reported in South Korea [31], which emphasizes increasing importance of assessing genetic diversity and population structure in this area. Since results obtained in this study strongly suggest an increase in the genetic diversity of *P. vivax *population in South Korea, monitoring of the parasite genetic diversity in this country as well as in neighbouring endemic areas will provide valuable information for developing malaria control strategies in South Korea.

## Conclusions

The present microsatellite analysis showed a recent expansion in the genome-wide genetic diversity of the *P. vivax *population in South Korea. This expansion is suggested to be due to recent migrations of different genotypes from other geographic areas into South Korea. Drastic change of *P. vivax *population structure during the period was also detected, in which a substantial increase in outbreeding partly contributes to the increased genetic diversity. Compared with genetic diversity in *P. vivax *populations from other geographic areas, the genetic diversity in South Korea is still low, although significant increase has been noted in recent years. This suggests that control of vivax malaria in this country could be achieved in line with WHO's agenda for malaria eradication.

## Competing interests

The authors declare that they have no competing interests.

## Authors' contributions

HH performed experiments, data analyses and paper writing. JYK and WL coordinated the sampling. NMQP, TM, JYK and TH critically reviewed the manuscript. TM, JYK, WL and KT participated in acquisition of funding. KT made substantial contributions to conceive the study design, paper writing and reviewing. All authors read and approved the final manuscript.

## Supplementary Material

Additional file 1 **Primers for PCR and sequencing of 13 microsatellite loci of *P. vivax***. Primer sequences for PCR amplification and direct sequencing of microsatellite loci are detailed. Forward and reverse primers are designated with "F" or "R", respectively. Click here for file

Additional file 2 **Allele frequency in 13 microsatellite loci of *P. vivax *populations in South Korea**. The alleles for each locus and their frequencies in two sample groups are presented. An allele for each locus corresponds to the number of repeat-motifs. Click here for file
