# Supplementary material for: Recent increase of genetic diversity in Plasmodium vivax population in the Republic of Korea
Source: Malar J. 2011 Sep 7;10:257. doi: 10.1186/1475-2875-10-257 (PMC3176257; doi:10.1186/1475-2875-10-257)
Supplement: Additional file 1 — Primers for PCR and sequencing of 13 microsatellite loci of P. vivax. Primer sequences for PCR amplification and direct sequencing of microsatellite loci are detailed. Forward and reverse primers are designated with "F" or "R", respectively. [file 1475-2875-10-257-S1.PDF]

Additional file 1. Primers for PCR and sequencing of 13 microsatellite loci of *P. vivax*

Locus	Chromosome	Motif	Used for	Sequence (5′-3′)	Reference
1.501	1	GGTGAGA	PCR	F: CCACGTGGGGCACCTTGTGATCGGGAAACA	13
			PCR	R: AGGCAGCGTTGGCGCAGTTGGAAGGGTTGA	
			sequencing	F: TGTTTCTTCCGCCCCGGCATT	
3.27	3	AAAC	PCR	F: TGGTCAGTTGGGTGGGACTACGTAGGGGT	13
			PCR	R: TCGCGCATCTGTGCACATTCGCTCGTTT	
			sequencing	F: ACACTCACCATGGCCGCTACA	
3.502	3	AACGGATG	PCR	F: TTGCCCCTTCACGCCCAGTGCACCCCTA	13
			PCR	R: CATTATACGCATCGATGTAACAGCAGCA	
			sequencing	F: CCATCAACTTGCATCTGCCGT	
MS3	4	GAA	PCR	F: AGCGCTCCCGAAACGAGACCGCAAATGT	17
			PCR	R: TGCCACTGCGAAGGTGGTGATTCCCTGGCA	
			sequencing	F: AAGCAGTGGAAGAGCGGAACGCCGTGGA	
MS15	5	TCT	PCR	F: TTGCTACTCGATCGGCTGTCACCTTTCA	17
			PCR	R: GACCTGAAAAAGGAGCAACAGCCAGACT	
			sequencing	F: TGTTGTTTTCCCCCTTTAGGT	
MS5	6	C(C/T)T	PCR	F: TTCTGCCCCTTCCGTTGCTCTCCTCGTCCA	17
			PCR	R: TGGGTGGAGTCCCATTTGGGGCACTGGCAT	
			sequencing	R: AAAGTAAGTCCCTAGACAGAGCCA	
MS9	8	GGA	PCR	F: TCTGAATTTCCCCATTTGCCCGTTTGGT	17
			PCR	R: CTTCTTCGGGTACCCCAGTATCACCTCT	
			sequencing	F: GCGAACAACATGAGCTTCACA	
MS16	9	(A/G)(C/T)(A/C)	PCR	F: TTGAGCATCGCCAAAGGGGTGGAGAGCGA	17
			PCR	R: TGTGTTCCTCCGCGCTGTTACGGCACCTT	
			sequencing	F: TTGCCTGAACAACGGCAGCGCA	
MS20	10	GAA	PCR	F: TTCACGCTGAAGGCCCCGAACGTAGGGAA	17
			PCR	R: CGCTCTTCATGTGGCAGTGGCTCATCTTCT	
			sequencing	F: ACTTGCTACTGCTGCCAAAGATG	
MS6	11	TCC	PCR	F: CATCGACGCTGCTTTGGTTTGGCAGAGCGT	17
			PCR	R: AGCAGTTGAAGCGGTGGGCCTTGCACGAT	
			sequencing	F: TCGGTGATCTCTGCGGTGGT	
MS8	12	CAA	PCR	F: TGAGGAGGGCGCCGGAGATAGCATTAGT	17
			PCR	R: AATTCATCACGTGGGGATACCTGCAACA	
			sequencing	F: CAGAAATGCAGAAGCAGAGGA	
MS10	13	A(A/G)(G/A)	PCR	F: TGCGGTGAACGAAGAGGACCAAACGGAGGA	17
			PCR	R: TGCACATTTGCGTCACCTCGGCGGGAGTTT	
			sequencing	F: ACGTCTATTACGCCTACGAGCA	
14.297	14	AAG	PCR	F: AGGCAAAGGGTAAAGCAGGAGCACGCGGA	13
			PCR	R: TGTGGCAACGAGCACTGGAGAGTTGACCT	
			sequencing	R: TCGCGTTGTTTTCTACCTCCGT	
