# Supplementary material for: Recent increase of genetic diversity in Plasmodium vivax population in the Republic of Korea
Source: Malar J. 2011 Sep 7;10:257. doi: 10.1186/1475-2875-10-257 (PMC3176257; doi:10.1186/1475-2875-10-257)
Supplement: Additional file 2 — Allele frequency in 13 microsatellite loci of P. vivax populations in South Korea. The alleles for each locus and their frequencies in two sample groups are presented. An allele for each locus corresponds to the number of repeat-motifs. [file 1475-2875-10-257-S2.PDF]

Additional file 2. Allele frequency in 13 microsatellite loci of *P. vivax* populations in South Korea

			1997–2000		2007	
Chromosome	Locus	Allele	*n*	Frequency	*n*	Frequency
1	1.501	3	16	0.55	26	0.90
		5	12	0.41	3	0.10
		11	1	0.03	0	0
3	3.27	7	1	0.03	0	0
		14	0	0	12	0.41
		17	0	0	12	0.41
		19	11	0.38	2	0.07
		20	17	0.59	3	0.10
3	3.502	4	11	0.38	5	0.17
		5	0	0	10	0.34
		11	0	0	9	0.31
		13	18	0.62	5	0.17
4	MS3	7	0	0	2	0.07
		8	1	0.03	0	0
		9	28	0.97	27	0.93
5	MS15	9	1	0.03	0	0
		11	11	0.38	2	0.07
		12	17	0.59	12	0.41
		13	0	0	11	0.38
		33	0	0	1	0.03
		34	0	0	3	0.10
6	MS5	9	0	0	3	0.10
		12	18	0.64	4	0.14
		14	0	0	5	0.17
		16	0	0	14	0.48
		17	10	0.36	1	0.03
		18	0	0	2	0.07
8	MS9	5	1	0.03	0	0
		6	10	0.34	23	0.79
		7	0	0	3	0.10
		8	18	0.62	3	0.10
9	MS16	21	12	0.43	8	0.28
		24	0	0	4	0.14
		44	1	0.04	0	0
		63	15	0.54	2	0.07
		66	0	0	10	0.34
		82	0	0	5	0.17
10	MS20	5	0	0	5	0.17
		6	28	0.97	24	0.83
		11	1	0.03	0	0
11	MS6	2	0	0	3	0.10
		6	0	0	12	0.41
		7	17	0.61	2	0.07
		9	1	0.04	7	0.24
		10	10	0.36	5	0.17
12	MS8	10	28	0.97	17	0.59
		26	1	0.03	0	0.00
		54	0	0	2	0.07
		55	0	0	8	0.28
		56	0	0	1	0.03
		58	0	0	1	0.03
13	MS10	32	1	0.03	0	0
		38	28	0.97	17	0.59
		39	0	0	12	0.41
14	14.297	9	0	0	1	0.03
		10	16	0.55	9	0.31
		12	12	0.41	3	0.10
		13	0	0	15	0.52
		14	0	0	1	0.03
		15	1	0.03	0	0
